# The Level of School Teachers’ Knowledge About First-Aid Management and Control of Epistaxis in Qassim Region, Saudi Arabia

**DOI:** 10.7759/cureus.33784

**Published:** 2023-01-15

**Authors:** Sultan Alanazy, Ibrahim Alqunibut, Rand Albahli, Laila Adawi, Maryam Aldehami, Ghaida Alharbi, Hind Alharbi, Rehana Khalil, Osama Al-Wutayd

**Affiliations:** 1 Department of Surgery, Ears, Nose, and Throat Unit, Unaizah College of Medicine and Medical Sciences, Qassim University, Unaizah, SAU; 2 Department of Medicine, Research Unit, Unaizah College of Medicine and Medical Sciences, Qassim University, Unaizah, SAU; 3 Department of Family and Community Medicine, Unaizah College of Medicine and Medical Sciences, Qassim University, Unaizah, SAU

**Keywords:** school teachers, saudi arabia, qassim, knowledge, epistaxis

## Abstract

Background: Epistaxis is an acute episode of nasal bleeding commonly caused in children by traumatic injuries in a school setting. It is one of the common ear, nose, and throat emergencies, which should be managed with first-aid measures. To the best of our knowledge, no studies have been conducted among school teachers in the Qassim region of Saudi Arabia regarding this information. This study thus aimed to assess levels of knowledge about first-aid management and control of epistaxis among school teachers in the Qassim region, Saudi Arabia.

Materials and methods: A cross-sectional study using a validated online questionnaire was distributed via social media platforms. Information was collected regarding sociodemographic characteristics, and eight items assessed participants’ knowledge about epistaxis and its management. Univariate, bivariate, and multivariable analyses were conducted to assess the factors associated with good levels of knowledge.

Results: The study had a total of 1,152 participants, of which 69.7% were female. The mean of knowledge was 3.29 (SD=1.39, range: 0-7). Only 19.4% of participants had a good level of knowledge. In multivariate analysis, females and those who had received information on first aid to stop nose-bleeds were significantly associated with good knowledge levels (adjusted odds ratio {AOR}: 1.72, 95% CI: 1.18-2.51, p=0.005; and AOR: 3.38, 95% CI: 2.47-4.64, p<0.001, respectively).

Conclusion: Less than one-quarter of participants had good knowledge levels. Health education sessions for teachers are highly recommended and should specifically target male teachers.

## Introduction

Epistaxis is a common ear, nose, and throat (ENT) pediatric emergency [1]. About 64% of epistaxis cases are seen in the age group of 11-15 years, while 56% in the age group of six to 10 years and 30% in the age group of zero to five years [2,3]. In 90% of cases, pediatric recurrent epistaxis originates from the anterior inferior part of the nasal septum known as Little’s area (Kiesselbach’s plexus) [4]. This anterior nosebleed is mostly trouble-free that is manageable, self-limiting, and only rarely requires medical intervention [5]. It is usually attributed to localized trauma from digital manipulation, local inflammation due to upper respiratory tract infection or mucosal drying, repeated common colds, and the use of steroid nasal sprays over a long period for the treatment of allergies [6-9]. Even so, non-accidental injury or serious illness could be the cause in the initial two years of life [10]. School is one of the main locations of pediatric epistaxis because children can have unintentional injuries while playing [11]. School teachers are responsible for the safety and well-being of the students at school [12]. It can be effectively managed at schools with simple measures such as digital compression on the nose and tilting the head forward [13]. In order for school teachers to deal with epistaxis in a timely and appropriate manner, awareness and knowledge of the correct first-aid measures are crucial [14-16]. However, it is found that there is limited or poor knowledge and practice of appropriate first-aid measures among school teachers, who are usually the first responders to encounter pediatric epistaxis at schools [16-20]. Evidence shows that factors that affect the knowledge of first aid among school teachers include age and educational level of the teacher, teaching experience, previous training on first aid, and their exposure to children in need of first aid [17,20].

As far as we know, no research studies have been conducted regarding risk factors or prevalence of epistaxis in Qassim region. However, there were two reasons which compelled us to conduct this study. Firstly, the first author (ENT specialist) found epistaxis is very common among school-age children of this region and is associated with allergic rhinitis. Secondly, a recent study conducted in 2022 among children aged six to 18 years estimated highest prevalence of allergic rhinitis as 51.3% in Qassim compared to other regions of Saudi Arabia [21]. Topical intranasal steroid spray is frequently used to treat allergic rhinitis, and recurrent epistaxis is the commonest side effect of this spray [22,23]. To the best of our knowledge, no study on this topic has been conducted among school teachers in the Qassim region to date. Therefore, our study aimed to assess the level of awareness about first-aid management and the control of epistaxis among school teachers in the Qassim region of Saudi Arabia.

## Materials and methods

Study design and setting

This web-based cross-sectional and non-representative study was conducted among teachers in Saudi Arabia between April 20, 2021, and October 20, 2021.

Questionnaire description

An Arabic-language questionnaire written according to the research objectives was used for this study [24]. It consisted of items collecting demographic characteristics of the teachers (age, gender, type of school, teaching level, and the teacher’s education level), along with eight items related to knowledge of epistaxis and its management. The questionnaire was scored by assigning one point for each correct answer and zero for incorrect ones. Participants who answered at least 60% correctly were classified as having good knowledge, and those with <60% were classified as having poor knowledge [25]. A pilot study was conducted with 25 teachers, and the results from the pilot were not included in this study.

Data collection

The questionnaire was accessible via Google Forms. The first page included the study’s title, its purposes and objectives, a declaration of confidentiality and anonymity, the voluntary nature of participation, how long it would take to complete the questionnaire (4-5 minutes), and the inclusion criteria (teachers currently in the Qassim region).

Sample size calculation

The minimum sample size was 359, as determined with OpenEpi software, assuming a 5% margin of error, confidence interval (CI) of 95%, and a level of awareness among teachers at 37.4%, based on a study conducted in Saudi Arabia [25].

Statistical analyses

Data were analyzed using STATA software version 16 (StataCorp LLC, College Station, Texas). The data are presented as a number (%) for categorical variables and as mean with standard deviation (SD) for continuous data. Bivariate analysis was conducted using the level of knowledge as the dependent variable and sociodemographic information as the independent variable. The crude odds ratio (COR) and 95% CI were reported. Multivariable analysis was performed using the level of knowledge as the dependent variable, and variables with a p<0.25 in the bivariate analysis as the independent variables. The adjusted odds ratio (AOR) and 95% CI were reported. A p<0.05 was considered strong evidence against the null hypothesis.

Ethical considerations

Ethical approval was received from the Ethics Committee of Qassim University (reference no. 20-07-05). All procedures performed in the study were in accordance with the ethical standards of institutional and national research committees and with the 1964 Helsinki Declaration and its later amendments. Online informed consent was obtained from each participant before their participation in the study.

## Results

A total of 1,152 participants were enrolled in the study. Out of that number, 509 (44.2%) participants were aged 36-45 years, more than half (69.7%) were females, 1,059 (91.9%) worked in governmental schools, 495 (43%) worked in elementary schools, 839 (72.8) had a bachelor’s degree, 688 (59.7%) had never received information about first aid to stop nose-bleeds or hemorrhage, and 631 (54.8%) had students in their classes who had episodes of epistaxis (Table 1). The participants’ knowledge of first-aid management and control of epistaxis was assessed with the eight items presented in Table 2. The mean knowledge score was 3.29 (SD=1.39, range: 0-7), and the correct responses ranged from 3.6% in response to “how long do you press on the nose?” to 75% correct answers in response to “will you try to stop bleeding by changing the head position?” Less than one-quarter of the participants (19.4%) had good knowledge scores.

**Table 1:** Demographic characteristics of study participants (n=1,152).

Characteristic		Number	%
Age (years)	≤25	90	7.8
	26-35	215	18.7
	36-45	509	44.2
	>45	338	29.3
Gender	Male	349	30.3
	Female	803	69.7
School type	Governmental	1059	91.9
	Private	93	8.1
Teaching level	Kindergarten	33	2.9
	Elementary school	495	43
	Secondary school	274	23.8
	High school	350	30.4
Education level	Diploma	242	21
	Bachelor’s degree	839	72.8
	Postgraduate degree	71	6.2
Have you ever received information about first aid to stop nosebleeds or hemorrhage?	Yes	464	40.3
	No	688	59.7
Have any of your students had epistaxis before?	Yes	631	54.8
	No	521	45.2

**Table 2:** Participants’ knowledge levels regarding first-aid management and control of epistaxis.

Statement	Options	Determination/score	n (%)
1. If you experience bleeding, what do you do?	Try to stop the bleeding	Correct/1	714 (62)
	Ask for help to stop the bleeding	Incorrect/0	351 (30.5)
	Call for an ambulance	Incorrect/0	87 (7.6)
2. If you experience bleeding, where do you press on the nose?	Upper part of nose (on the bone)	Incorrect/0	615 (53.4)
	Bottom of the nose (on the cartilage)	Correct/1	306 (26.6)
	I don’t know	Incorrect/0	231 (20.1)
3. How long do you press on the nose?	<5 minutes	Incorrect/0	343 (29.8)
	5-10 minutes	Incorrect/0	315 (27.3)
	>10 minutes	Correct/1	42 (3.6)
	Until bleeding stops	Incorrect/0	199 (17.3)
	I don’t know	Incorrect/0	253 (22)
4. Will you try to fill the nose with a tissue or gauze?	Yes	Incorrect/0	511 (44.4)
	No	Correct/1	641 (55.6)
5. Will you try to stop the bleeding by changing the head position?	Yes	Correct/1	864 (75)
	No	Incorrect/0	288 (25)
6. How do you change the head position?	Tilt it forward	Correct/1	564 (49)
	Tilt it backward	Incorrect/0	451 (39.1)
	I don’t know	Incorrect/0	137 (11.9)
7. Will you try to put ice on your head or nose?	On the head	Incorrect/0	390 (33.9)
	On the nose	Correct/1	328 (28.5)
	Chew the ice	Incorrect/0	28 (2.4)
	I will not use ice	Incorrect/0	406 (35.2)
8. When do you think you should go to an emergency department?	I will not call for emergency	Incorrect/0	65 (5.6)
	If bleeding lasts >10 minutes	Incorrect/0	610 (53)
	If bleeding lasts >30 minutes	Correct/1	331 (28.7)
	If bleeding lasts >60 minutes	Incorrect/0	74 (6.4)
	I don’t know	Incorrect/0	72 (6.3)

In bivariate analysis, the age group of 26-35 years was negatively associated with good knowledge (OR: 0.57, 95% CI: 0.36-0.89, p=0.014), female participants were significantly associated with good knowledge levels (OR: 2.07, 95% CI: 1.45-2.97, p<0.001), and those who received information about first aid to stop nosebleeds were significantly associated with good levels of knowledge (OR: 3.77, 95% CI: 2.77-5.13, p<0.001) (Table 3). The variables that maintained significance in the multivariable analysis were being female and receiving information about first aid for nosebleeds, both of which were significantly associated with good knowledge scores (AOR: 1.72, 95% CI: 1.18-2.51, p=0.005; and AOR: 3.38, 95% CI: 2.47-4.64, p<0.001, respectively) (Table 4).

**Table 3:** Bivariate analysis of factors associated with good levels of knowledge. *Not included in multivariable analysis. COR: crude odds ratio

Characteristic		Good knowledge, n (%) 224 (19.4)	Poor knowledge, n (%) 928 (80.6)	COR (95% CI)	p-Value
Age	≤25	26 (28.9)	64 (71.1)	1.54 (0.93-2.56)	0.091
	26-35	28 (13)	187 (87)	0.57 (0.36-0.89)	0.014
	36-45	106 (20.8)	403 (79.2)	Reference	-
	>45	64 (18.9)	274 (81.1)	0.89 (0.63-1.26)	0.501
Gender	Male	43 (12.3)	306 (87.7)	Reference	-
	Female	181 (22.5)	622 (77.5)	2.07 (1.45-2.97)	<0.001
Education level*	Diploma	51 (21.1)	191 (78.9)	1.12 (0.78-1.59)	0.543
	Bachelor’s degree	162 (19.3)	677 (80.7)	Reference	-
	Postgraduate degree	11 (15.5)	60 (84.5)	0.77 (0.39-1.49)	0.433
Teaching level	Kindergarten	11 (33.3)	22 (66.7)	2.05 (0.96-4.37)	0.063
	Elementary school	97 (19.6)	398 (80.4)	Reference	-
	Intermediate school	47 (17.2)	227 (82.9)	0.85 (0.58-1.24)	0.406
	Secondary school	69 (19.7)	281 (80.3)	1.01 (0.71-1.42)	0.966
School type	Governmental	201 (19)	858 (81)	Reference	-
	Private	23 (24.7)	70 (75.3)	1.40 (0.85-2.30)	0.181
Have you ever received information about first aid to stop nosebleeds or hemorrhages?	No	76 (11.1)	612 (88.9)	Reference	-
	Yes	148 (31.9)	316 (68.1)	3.77 (2.77-5.13)	<0.001
Have any of your students had epistaxis before?	No	91 (17.5)	430 (82.5)	Reference	-
	Yes	133 (21.1)	498 (78.9)	1.26 (0.94-1.69)	0.124

**Table 4:** Multivariable analysis of factors associated with good knowledge. AOR: adjusted odds ratio

Characteristic		AOR	95% CI	p-Value
Age (years)	≤25	1.26	0.72-2.23	0.419
	26-35	0.67	0.41-1.09	0.113
	36-45	Reference	-	-
	>45	0.84	0.58-1.21	0.345
Gender	Male	Reference	-	-
	Female	1.72	1.18-2.51	0.005
Teaching level	Kindergarten	1.48	0.64-3.43	0.359
	Elementary school	Reference	-	-
	Intermediate school	0.94	0.63-1.42	0.794
	Secondary school	1.07	0.73-1.56	0.720
School type	Governmental school	Reference	-	-
	Private school	1.26	0.71-2.27	0.429
Have you ever received information about first aid to stop nosebleeds or hemorrhage?	No	Reference	-	-
	Yes	3.38	2.47-4.64	<0.001
Have any of your students had epistaxis before?	No	Reference	-	-
	Yes	1.23	0.89-1.68	0.200

## Discussion

This study’s results showed that 80.6% of our participants in the Al Qassim region had low levels of knowledge about first aid in the management of epistaxis among school children. That result is comparable with a recent study conducted in the Asser region of Saudi Arabia, which reported that 84.5% of teachers had poor levels of knowledge about the topic [26]. Conversely, Abdulsalam et al. in 2021 reported 66.0% of school teachers had low levels of knowledge [27]. Other studies with similar objectives conducted in Saudi Arabia, including the Riyadh region (2015), Taif region (2021), and Abha city (2019), showed comparatively lower proportion of poor knowledge among teachers than our participants [25,28,29]. There were variations in the results of studies conducted in Sudan, Palestine, and Turkey, which showed low knowledge levels among 63.3%, 33.6%, and 65.1% of respondents, respectively [30-32].

In the current study, more than one-third of the study participants had previously received information about first aid for the control of epistaxis, which is similar to a study conducted in the Aseer region of Saudi Arabia that reported 45.2% had previously received information about first aid [26]. However, the result in our study is lower than other Saudi studies that claimed 54.5%, 68%, 94%, and 56% of their respondents had previously received information [24,25,27,28]. Our results were that about half of our participants had taught students with epistaxis during their careers, which is higher than the results of an online study conducted in Saudi Arabia, which found 41.9% had encountered epistaxis in students, but it was lower than the experience of study participants from other regions of Saudi Arabia, including Riyadh (68.1%), Al Ahsa (67%), Aseer (72.6%), and Taif (66.6%) [24-28]. Almost two-thirds of our participants were aware that with epistaxis, they must try to stop the bleeding, which is a lower percentage than in the studies conducted by Alshehri et al., which showed 68% of their participants knew this and Al-Kubaisy et al., which reported 76.5% of their participants were aware of this fact [24,25].

Additionally, one-quarter of our participants knew that they must refer the case to the emergency department if bleeding lasts longer than 30 minutes. This finding is consistent with findings from Abdulsalam et al., which reported 23.8% in this regard [27] while studies by Alasiri et al. and Aljuaid et al. showed higher awareness levels, with 31.6% and 32.6%, respectively [26,28]. In the present study, one-quarter of the participants were aware that first aid for epistaxis includes the application of compression on the cartilaginous part of the nose, but only a few (3.6%) knew the correct compression time of more than 10 minutes. This finding is in agreement with other Saudi studies that showed 23% and 25% awareness levels, respectively, but their reported levels of knowledge about compression time were a bit higher than ours at 12.8% and 7%, respectively [24,25]. Other studies have reported 27.4%, 30%, 29.1%, and 64.6% of participants had awareness of compression on the cartilaginous part of the nose, and the correct compression time was reported among 11.9% and 10.1% of participants in other studies [26,28,33,34]. 

One-quarter of our participants correctly stated that they would put ice on the nose, which is a much lower percentage than other Saudi studies that had 57.8%, 57.4%, 55.8%, and 53% correct responses but is higher than an Ethiopian study (18.5%) [24-26,28,33]. More than half of our respondents responded correctly that they would not fill the nose with tissue or gauze. This is similar to findings reported by Al-Kubaisy in Riyadh (55.9%) and Alasiri et al. in the Aseer region (50.5%) [25,26]. Additionally, our rate is lower than studies by Alshehri et al. (63.5%) and Aljuaid et al. (61.3%), but higher than a study by Abdulsalam et al. (45.5%) [24,27,28]. In the present study, three-quarters of participants reported that they would try to change the position of the head, but only half of the participants knew they should tilt the head forward. This finding is more or less in agreement with other studies that demonstrated rates of 84.9% (Taif) and 78.3% (Riyadh) for awareness of changing the position of the head [25,28]. A similar range of results have been reported about tilting it forward, with 57% (Al Ahsa), 60.2% (Riyadh), 58.1% (Aseer), 49.9% (Taif), and 50.8% (Hail) [24-26,28,34]. However, the Ethiopian study revealed a higher rate of 60% of respondents aware that the head should be tilted forward [33].

In multivariable analysis, being female and having previously received information about first aid to stop nosebleeds were associated with good knowledge levels. Previous research has also shown that having good knowledge of first aid for epistaxis was significantly associated with having previously received information on the topic, as well as practicing and training on epistaxis first aid [25,26,28,35]. Unlike Abdulsalam et al., we found no significant association between good knowledge levels and a participant’s age [27]. Interestingly, no other study reported an association between good knowledge levels and being female. A possible explanation for this could be the child-rearing experience of the female teachers as mothers, who may also have their own children suffering from epistaxis at home. However, it needs to be investigated through further research studies.

This study has numerous limitations. We cannot establish a causal relationship due to the cross-sectional study design, and our data represent a single time point. Another limitation is related to non-probability sampling for this study, as the participants were included through the researchers’ networks and disseminated via social media platforms. Future studies with representative samples are required to strengthen the findings of this study. Further research to propose the prevention and management modules or strategies for common causes of epistaxis is highly recommended to reduce its incidence among school-age children.

## Conclusions

School teachers’ awareness of the management and control of pediatric epistaxis is far below optimum levels. Our findings support the notion that school teachers should be trained in first-aid management and control of epistaxis to ensure that their skills are up-to-date for practical application. This can be achieved by incorporating first-aid courses for teachers as a part of teacher training programs to get teaching license and providing free courses for teachers held in their schools at regular intervals.
